# Supplementary material for: MiRNA Profiling in Plasma and Placenta of SARS-CoV-2-Infected Pregnant Women
Source: Cells. 2021 Jul 15;10(7):1788. doi: 10.3390/cells10071788 (PMC8305278; doi:10.3390/cells10071788)
Supplement: Supplementary file 1 [file cells-10-01788-s001.zip › cells-1264886-supplementary.pdf]

Supplementary Table 1. Timing of infection and leukocyte distribution in SIPW and UPW

SARS-CoV-2 Infected Pregnant Women (SIPW)	Δ (in days)*	Leukocyte distribution**				
		Neutrophils	Lymphocytes	Monocytes	Eosinophils	Basophils
1	1	9,15	2,1	1,08	0,48	0,03
2	0	9,44	1,1	0,41	0	0,01
3	1	12,44	0,38	1	0,13	0,03
4	3	5,58	1,14	0,47	0	0,01
5	7	4,57	1,05	0,35	0,02	0,01
6	1	3,18	0,77	0,42	0	0,01
7	6	8,15	0,87	0,82	0,03	0,03
8	1	6,5	0,82	0,55	0,01	0,01
9	-1	17,69	3,07	1,54	0,68	0,1
10	2	7,17	2,17	0,67	0,07	0,02
11	0	11,46	1,54	1,04	0,08	0,03
12	1	5,98	1,04	0,51	0,01	0,01
13	3	10,27	2,55	0,72	0,12	0,07
14	0	9,48	1,7	0,43	0,01	0,02
15	9	7,83	2,94	0,74	0,08	0,04

Uninfected Pregnant Women (UPW)	Δ (in days)*	Leukocyte distribution **				
		Neutrophils	Lymphocytes	Monocytes	Eosinophils	Basophils
1	0	N/A	N/A	N/A	N/A	N/A
2	0	N/A	N/A	N/A	N/A	N/A
3	0	6,19	2,46	0,95	0,35	0,07
4	0	11,87	2,28	1,43	0,17	0,03
5	0	5,93	2,81	0,77	0,08	0,03
6	0	7,21	2,76	0,76	0,13	0,04

* Difference in days between the first SARS-CoV-2 positive nasopharyngeal swab and day of delivery.

** Absolute cell count, cell x 10^3/µl of blood.
